# Acquisition of cross-azole tolerance and aneuploidy in *Candida albicans* strains evolved to posaconazole

**DOI:** 10.1093/g3journal/jkac156

**Published:** 2022-07-26

**Authors:** Rebekah J Kukurudz, Madison Chapel, Quinn Wonitowy, Abdul-Rahman Adamu Bukari, Brooke Sidney, Riley Sierhuis, Aleeza C Gerstein

**Affiliations:** Department of Microbiology, The University of Manitoba, Winnipeg, MB R3T 2N2, Canada; Department of Microbiology, The University of Manitoba, Winnipeg, MB R3T 2N2, Canada; Department of Microbiology, The University of Manitoba, Winnipeg, MB R3T 2N2, Canada; Department of Microbiology, The University of Manitoba, Winnipeg, MB R3T 2N2, Canada; Department of Microbiology, The University of Manitoba, Winnipeg, MB R3T 2N2, Canada; Department of Microbiology, The University of Manitoba, Winnipeg, MB R3T 2N2, Canada; Department of Microbiology, The University of Manitoba, Winnipeg, MB R3T 2N2, Canada; Department of Statistics, The University of Manitoba, Winnipeg, MB R3T 2N2, Canada

**Keywords:** drug resistance, drug tolerance, aneuploidy, experimental evolution

## Abstract

A number of in vitro studies have examined the acquisition of drug resistance to the triazole fluconazole, a first-line treatment for many *Candida* infections. Much less is known about posaconazole, a newer triazole. We conducted the first in vitro experimental evolution of replicates from 8 diverse strains of *Candida albicans* in a high level of the fungistatic drug posaconazole. Approximately half of the 132 evolved replicates survived 50 generations of evolution, biased toward some of the strain backgrounds. We found that although increases in drug resistance were rare, increases in drug tolerance (the slow growth of a subpopulation of cells in a level of drug above the resistance level) were common across strains. We also found that adaptation to posaconazole resulted in widespread cross-tolerance to other azole drugs. Widespread aneuploidy was observed in evolved replicates from some strain backgrounds. Trisomy of at least one of chromosomes 3, 6, and R was identified in 11 of 12 whole-genome sequenced evolved SC5314 replicates. These findings document rampant evolved cross-tolerance among triazoles and highlight that increases in drug tolerance can evolve independently of drug resistance in a diversity of *C. albicans* strain backgrounds.

## Introduction

Drug resistance is a critical threat to global public health. Antimicrobial resistance is inherently an evolutionary phenomenon: drug-resistant individuals arise and spread within susceptible populations. The genetic basis and rate of adaptation in a drug is in part a deterministic process, akin to the evolutionary process under any environmental stress, influenced by the specifics of the environment, the microbial population size, the mutation rate, and the effect size of available beneficial mutations. Unlike bacteria, which frequently acquire plasmid-mediated beneficial genes and alleles from the environment, fungal microbes primarily adapt via vertical transmission. Genomic variation within evolving populations often includes small-scale point mutations and insertions and deletions as well as larger-scale karyotypic mutations in ploidy (the number of chromosome sets), aneuploidy (copy number change in one or several chromosomes), and zygosity (the number of alleles at a given position in the genome; Selmecki *et al.* 2015; Ene *et al.* 2018, 2021; Wang *et al.* 2018). Few antifungal drug classes are currently approved for the treatment of fungal infections. One strategy to prolong the utility of existing drugs is to better understand the factors that influence fungal resistance acquisition, to reduce the likelihood that resistance will arise.

In addition to drug resistance, which is often measured as the minimum inhibitory concentration (MIC) of drug that reduces growth by some amount (e.g. 50% or 80%) after 24 h, drug tolerance has recently emerged as an important parameter in characterizing drug response in fungal species. Fungal tolerance (which is distinct from bacterial tolerance; Levin-Reisman *et al.* 2017) is defined as the proportion of the population that grows slowly in drug concentrations above the MIC (Rosenberg *et al.* 2018; Berman and Krysan 2020). Although few studies have quantified tolerance yet, it may play a role in persistent candidemia (Rosenberg *et al.* 2018) and mortality (Levinson *et al.* 2021). From an evolutionary perspective, beneficial mutations that arise in populations evolving in fungistatic environments (that primarily inhibit rather than kill susceptible cells) could act in two distinct pathways: they can increase resistance (i.e. increase the MIC), or they can increase tolerance (i.e. enable a larger proportion of the population to grow above the MIC). A recent screen of 235 clinical *Candida* spp. isolates found that resistant isolates also tended to be more tolerant to the fungistatic drug fluconazole (FLC; Salama and Gerstein 2022). However, a large-scale in vitro evolution experiment in FLC found that changes in tolerance evolved independently of resistance (Gerstein and Berman 2020). As many antifungal drugs are fungistatic rather than fungicidal, increasing drug tolerance may be an unappreciated yet critical selective avenue for fungal populations adapting to a drug.

Relatively few species account for most human fungal infections, with *Candida albicans* the primary species responsible for mucosal disease, *Aspergillus fumigatus* for allergic disease, and *Trichophyton* spp. for skin infections (Bongomin *et al.* 2017). The azole drugs FLC and voriconazole (VCZ) are first-line treatments for candidiasis and aspergillosis, respectively (Pappas *et al.* 2016; Patterson *et al.* 2016). Primarily through the study of FLC-resistant *C.*  *albicans* strains, the genetic basis of major adaptive pathways have been identified: the first involves alterations or overexpression of *ERG11*, which produces the target demethylase (Asai *et al.* 1999; Lamb *et al.* 2000; Selmecki *et al.* 2008; Flowers *et al.* 2015; Paul *et al.* 2019; Lee *et al.* 2021); the second is through the upregulation of drug efflux pumps encoded by *CDR1*, *CDR2*, *TAC1*, *MRR1*, and *MDR1* (Sanglard *et al.* 1995; Asai *et al.* 1999; Lamb *et al.* 2000; Coste *et al.* 2006; Selmecki *et al.* 2008; Flowers *et al.* 2015; Paul *et al.* 2019; Lee *et al.* 2021). One of the newest azoles, posaconazole (POS), effectively treated infections resistant to first-line azoles in *A.*  *fumigatus*, *C. albicans*, and *C. neoformans* (Chau *et al.* 2004; Xiao *et al.* 2004; Firinu *et al.* 2011; Sionov *et al.* 2012). Intriguingly, although specific point mutations in *ERG11* homologs confer POS cross-resistance to azoles and other antifungal drugs in *Aspergillus* spp. (Lockhart *et al.* 2011; D’Agostino *et al.* 2018; Abastabar *et al.* 2019) single* ERG11* point mutations do not provide the same degree of POS cross-resistance in *C. albicans* (MacCallum *et al.* 2010; Sanglard and Coste 2016; Warrilow *et al.* 2019). Rather, it seems that multiple *ERG11* mutations are required for POS resistance (Li *et al.* 2004). This may be attributable to the extended side chain of POS interacting with an additional domain of the target enzyme (Chau *et al.* 2004; Li *et al.* 2004; Xiao *et al.* 2004; Katragkou *et al.* 2012).

*Candida*  *albicans* is a predominantly diploid asexual organism. Genetic diversity within populations is primarily achieved through mitosis, though parasexual reproduction is also possible (Hickman *et al.* 2013; Ene and Bennett 2014). In addition to point mutations, unicellular fungal microbes seem prone to acquiring chromosomal aneuploidies during adaptation (Gerstein and Berman 2015; Gerstein and Sharp 2021) and mitotic recombination results in loss of heterozygosity (LOH). Intriguingly, exposure to the triazole drugs FLC, ketoconazole (KCZ), VCZ, and itraconazole potentiate the appearance of chromosomal aneuploidy (Harrison *et al.* 2014). Extra copies of hr3 (Perepnikhatka *et al.* 1999; Ford *et al.* 2015), Chr4 (Perepnikhatka *et al.* 1999; Anderson *et al.* 2017), and Chr5 (Coste *et al.* 2006; Selmecki *et al.* 2006, 2008; Ford *et al.* 2015; Todd *et al.* 2019) have previously been shown to confer increased resistance to FLC, hence azole drugs cause both a generalized increased rate of aneuploidy as well as selection for specific aneuploidies. Resistance has been attributed to increased gene dosage of drug transporters or their transcriptional activators [*CDR1*, *CDR2*, *CZR1*, and *MRR1* on Chr3 (Sanglard *et al.* 1995, 1997; Chau *et al.* 2004; Coste *et al.* 2004, 2006; Dunkel *et al.* 2008; Robbins *et al.* 2017; Todd and Selmecki 2020), and *TAC1* on Chr5 (Selmecki *et al.* 2008)], stress response proteins [*PBS2* on Chr3 (Todd and Selmecki 2020), and *CGR1* on Chr4 (Todd and Selmecki 2020)], and the target enzyme [*ERG11* on Chr5 (Chau *et al.* 2004; Selmecki *et al.* 2008)]. Furthermore, since many genes are affected by an aneuploidy, nontargeted effects may be more common than with single-gene mutations; for example, Chr2 aneuploidy selected under caspofungin exposure confers enhanced survival by different mechanisms to hydroxyurea (Yang *et al.* 2019) and tunicamycin (Yang, Gritsenko, *et al.* 2021). In some cases, this may provide an enhanced selective effect for aneuploidy to sweep through a population. In other scenarios, gene overexpression could be selectively disadvantageous (Yang, Todd, *et al.* 2021) reducing the potential fitness benefit. Whether consistent POS exposure also selects for beneficial aneuploidy has not been determined.

Here, we conducted the first in vitro experimental evolution of 8 diverse strains of *C.*  *albicans* in the fungistatic drug POS. We found that increases in drug tolerance to POS were common across strain backgrounds, while increases in drug resistance were rare. We also found that adaptation to POS resulted in widespread cross-tolerance to other azoles and widespread increases in genome size.

## Materials and methods

### Strains and evolution

Eight clinical strains of *C. albicans* from different phylogenetic clades that span ancestral resistance to FLC (Gerstein and Berman 2020) were selected: FH1 (clade 3, FLC MIC = 4, Fonzi and Irwin 1993), SC5314 (clade 1, FLC MIC = 0.5, Lockhart *et al.* 2002), T101 (clade 3, FLC MIC = 32, Odds *et al.* 2007), GC75 (clade 4, FLC MIC = 0.0125, Wu *et al.* 2007), P75016 (clade 4, FLC MIC = 0.5, Wu *et al.* 2007), P76055 (clade 2, FLC MIC = 0.0125, Wu *et al.* 2007), P78048 (clade 1, FLC MIC = 0.5, Wu *et al.* 2007), and P87 (clade 4, FLC MIC = 1, Wu *et al.* 2007). Freezer stock was streaked onto YPD agar plates, a standard lab yeast rich medium (2% w/v peptone, 2% w/v yeast extract, 1.8% w/v agar, 1% w/v glucose, 0.00016% w/v adenine sulfate, 0.00008% w/v uridine, and 0.1% v/v of each chloramphenicol and ampicillin), and incubated at room temperature for 72 h. Twelve single colonies from each of the 8 strains were haphazardly selected and transferred into 1 mL of liquid YPD in a 96 deep-well box, sealed with Breathe–Easier sealing membranes (Electron Microscopy Sciences, PA, USA), and incubated for 24 h at 30°C, creating 12 replicate lines from each strain. Each replicate was frozen down in triplicate in 15% glycerol and stored at −70°C as the ancestral culture.

Two sets of evolution experiments were similarly initiated. Optical density was measured from ancestral replicates grown in YPD, and the cultures were standardized to an OD_600_ of 0.01. A 1:10 dilution was then done in parallel into either YPD + 0.5 µg/mL POS (which we will refer to as POS0.5) or YPD alone. For the first set, replicate lines were initially incubated statically at 30°C for 24 h, followed by 1:1,000 serial dilutions into fresh medium (POS0.5 or YPD) every 24 h for 4 days, for a total of ∼50 generations of evolution. We chose to incubate statically to mimic the guidelines for clinical MIC testing (CLSI 2008). The second set of experiments followed a very similar method, except all replicates were initially incubated statically at 30°C for 72 h, and then four 1:1,000 serial dilutions were done into fresh POS0.5 medium every 72 h. Twelve replicates from strains P87, GC75, and SC5314 were evolved with both 24 and 72 h transfers in POS0.5 in a pilot study that followed the same protocol before the main study.

Evolved replicates were frozen down in triplicate in 15% glycerol after the fifth transfer and stored at −70°C. In total, 132 replicates were evolved for each transfer duration in POS [(12 replicates × 3 strains) + (12 replicates × 8 strains) = 132 replicates] while 96 replicates were evolved for each in YPD. Replicates were considered extinct at the end of the experiment if they were unable to be revived from the evolved freezer stock.

### MIC of ancestral strains

The 8 ancestral strains were assayed for growth in 10 drug concentrations that increased from 0.001 to 0.5 μg/mL. The MIC of POS was determined as the highest drug concentration where the OD_600_ after 24 h incubation was at least 50% of OD_600_ in the absence of drug. Freezer stock of ancestral strains was streaked onto YPD and incubated for 48 h at 30°C. Culture from each strain was inoculated into 500 µL of YPD in a 96 deep-well box, covered with a Breathe–Easy sealing membrane, and incubated shaking overnight at 30°C. A 1:1,000 dilution from overnight cultures was inoculated into 100 μL of each of the 10 drug concentrations and YPD (i.e. no drug control) in a round bottom plate. The plates were incubated statically at 30°C, with OD_600_ measurements taken from mixed wells at 24 h. Two biological and technical replicates were measured for each strain.

### Growth ability in the evolutionary environment

Growth ability in the evolutionary environment was measured as OD_600_ after 24 and 72 h incubation. Five microliters of thawed freezer stock was transferred from ancestral and evolved replicates into 500 mL liquid YPD and incubated at 30°C. After 48 h, culture from all replicates was standardized to OD_600_ 0.01 in liquid YPD. One hundred microliters of standardized culture was then placed into each well of a 96-well round bottom plate, and 100 µL of YPD + 1 µg/mL POS was added to each well (i.e. a final concentration of 0.5 µg/mL POS). Plates were covered with a Breathe–Easier sealing membrane and incubated statically at 30°C for 72 h, with OD_600_ measurements taken every 24 h.

### Drug susceptibility

Drug susceptibility was measured by disk diffusion assays in POS and FLC in all replicates. In addition, SC5314 replicates were also assayed in miconazole (MCZ), clotrimazole (CTR), VCZ, 5-fluorocytosine (5-FC), and nystatin (NYT). Posaconazole disks were prepared by adding 4 µL of 0.625 mg/mL POS stock in DMSO to blank susceptibility disks (Fisher Scientific, Ottawa, ON, Canada) for a final concentration of 2.5 mg. All other susceptibility disks were purchased: FLC (25 µg; Fisher Scientific, Ottawa, ON, Canada); MCZ (50 µg), CTR (50 µg), VCZ (1 µg), 5-FC (1 µg), and NYT (100 IU; BioRad Laboratories, Hercules, CA, USA).

Ancestral and evolved replicates were grown from frozen stocks in liquid YPD at 30°C for 48 h. Each replicate was standardized to OD_600_ 0.01. Next, 100 μL of standardized culture was spread, in duplicate, onto 15 mL YPD agar plates, using sterile 5-mm glass beads. A single drug susceptibility disk was applied to the center of each plate, and plates were incubated at 30°C. After 48 h, each plate was placed on a lightbox and photographed from above in a darkroom using a Canon EOS Rebel SL2.

Photographs were cropped, converted to 8-bit, inverted, and brightness and contrast were altered using a custom script in ImageJ (Schneider *et al.* 2012) to obtain bright colonies against a black background. Resistance (RAD_20_) and tolerance (FoG_20_) were quantified from the images using the *diskImageR* R package, following recommendations specified in the diskImageR vignette V2 (Gerstein *et al.* 2016; https://www.microstatslab.ca/diskimager.html; last accessed June 26, 2022). The reported RAD_20_ and FoG_20_ values are averages across multiple biological and technical replicates.

### Ploidy variation

Flow cytometry was used to determine if evolved replicates had altered ploidy (all were initially diploid). Ancestral and surviving evolved replicates were fixed, stained, and measured in parallel. Five microliters of each replicate was inoculated from frozen into 500 μL of liquid YPD in a 96 deep-well box, covered with a Breathe–Easier sealing membrane, and shaken at 350 rpm at 30°C for 48 h. After 48 h, 10 μL was subcultured into 500 μL of fresh media and shaken at 350 rpm at 30°C for 4 h. Two hundred microliters of subculture was then transferred to a 96-well round bottom plate and pelleted. Pellets were resuspended in 20 μL of 50:50 Tris-EDTA (TE), fixed by slowly adding 180 μL of 95% cold ethanol, and stored wrapped in aluminum foil at −20°C for at least 12 h.

The fixed culture was pelleted, washed in 200 μL of TE, pelleted again, and resuspended in 50 μL of 1 mg/mL RNAse A solution (New England Biolabs, Ipswich, MA, USA) and statically incubated at 37°C for 3 h. After the 3 h incubation, the replicates were pelleted and resuspended in 50 μL TE and 50 μL of 1:100 SYTOX: TE solution and incubated at room temperature in the dark overnight. The next day the replicates were pelleted then resuspended in 700 μL of TE. All centrifugation steps were done at 1,000 × g for 5 min.

Flow cytometry was performed on an SH800S Cell Sorter (Sony Biotechnology Inc., San Jose, CA, USA). All replicates had an event rate of 600–1,000 events/second and a total of 10,000 events were recorded. Data was uploaded to FlowJo (Tree Star, Ashland, OR, USA), and debris was excluded via gating. Each replicate population was then fit with the Watson (pragmatic) cell cycle algorithm (Watson *et al.* 1987) to determine the mean G1 peak.

### DNA extraction and variant calling

Genomic DNA was extracted from 2 ancestral strain replicates, and 12 evolved replicates from SC5314, the strain used for the *C. albicans* reference genome. Thirty microliters of each replicate were transferred into 3 mL of YPD and incubated shaking overnight at 37°C. The culture was centrifuged at 2,500 rpm for 3 min, and the supernatant was discarded. The pellet was resuspended in 500 μL of TENTS buffer (100 mM NaCl, 10 mM Tris pH 8.0, 1 mM EDTA, 2% Triton X 100, and 1% SDS), 100 μL of glass beads, and 200 μL phenol: chloroform: IAA and vortexed for 20 min at 4°C followed by centrifugation for 10 min at 13,500 rpm. After centrifugation, 350 μL of supernatant was transferred to a sterile microcentrifuge tube, and 1 mL of cold 100% ethanol was added and left overnight at −20°C to allow for DNA precipitation. This was followed by centrifugation at 13,500 rpm for 10 min, and the pellet was resuspended in 50 μL of molecular biology grade water. One microliter of 10 mg/mL RNAse A was added and incubated at 37°C for 1 h, after which 2 μL of 20 mg/mL proteinase K (Fisher Scientific, Ottawa, ON, Canada) was added and incubated at 37°C for 1.5 h. After incubation, 200 μL of molecular biology grade water and 300 μL of phenol: chloroform: IAA was added, and samples were centrifuged at 13,500 rpm for 10 min. Next, the supernatant was transferred to a sterile microcentrifuge tube, 4 μL of 5 M NaCl and 400 μL of cold 100% ethanol were added, and DNA was precipitated for at least 10 min at −20°C. Tubes were then centrifuged at 13,500 rpm for 10 min, the supernatant was discarded, and the pellet was left overnight. The DNA pellet was then resuspended in 40 μL of molecular biology grade water.

DNA quality was assessed using the Thermo Scientific NanoDrop 2000, and DNA concentration was measured using a Qubit 2.0 Fluorometer (following the Invitrogen Qubit dsDNA BR Assay Kit; Thermo Fisher Scientific, Waltham, MA, USA). Whole-genome sequencing was performed by the Microbial Genome Sequencing Center (Pittsburgh, USA) using the Illumina NextSeq 550 platform with paired-end reads of 2 × 150 bp. Reads were trimmed (Trimomatic v 0.39; Bolger *et al.* 2014) and mapped using bwa-mem (Li 2013) to the SC5314 reference genome (A21-s02-m09-r10). Average coverage was estimated using samtools (v 1.13; Li *et al.* 2009); mean depth among sequenced replicates (excluding the mitochondrial chromosome) was 83× (Supplementary Table 1).

To check for mutations in known antifungal resistance genes, the mapped reads were processed by removing duplicate PCR amplicons and fixing mate-pairs using picard (Broad Institute 2018). Base quality scores were recalibrated with known single-nucleotide polymorphisms discussed in Jones *et al.* (2004) obtained from the Candida Genome Database website (http://www.candidagenome.org/download/gff/C_albicans_SC5314/Assembly21/A21_Jones_PMID_15123810_Polymorphisms.vcf; downloaded on July 29 2020) using the BaseRecalibrator from the Genome Analysis Toolkit (McKenna *et al.* 2010). Genotypes were joint called and hard filtered [QualByDepth (QD) < 2.0, FisherStrand (FS) > 60.0, root mean square mapping quality (MQ) < 30.0, MappingQualityRankSumTest (MQRankSum) < −12.5, ReadPosRankSumTest (ReadPosRankSum) < −8.0] using Genome Analysis Toolkit’s HaplotypeCaller, CombineGVCFs, GenotypeVCFs, VariantFiltration, and SelectVariants (DePristo *et al.* 2011; Van der Auwera *et al.* 2013; Poplin *et al.* 2017) as previously done for *C.*  *albicans* (Ropars *et al.* 2018). The effect of each variant on gene function was predicted using the SnpEff program (v 5.1; Cingolani *et al.* 2012) using an inhouse database build of the A21-s02-m09-r10 reference. A visual inspection in Integrative Genomics Viewer (IGV v2.8.3; Thorvaldsdóttir *et al.* 2013) and a custom R script was then used to look for mutations within known antifungal resistance genes. Fastq files have been deposited in the National Center for Biotechnology Information Sequence Read Archive database as PRJNA813559. Shell and R scripts are available at https://github.com/acgerstein/posaconazole-evolution.

### Karyotype analysis

To analyze the whole-genome sequences for karyotypic variation in evolved SC5314 replicates, we used Y_MAP_, a computational pipeline that visualizes copy number variation (CNV) and LOH (Abbey *et al.* 2014). Mitochondrial DNA was excluded from the coverage analysis. Paired-end read data were uploaded and analyzed using the SC5314 A21-s02-m09-r07 reference genome. Both baseline and experimental ploidy were left at the default values (2, i.e. diploid), and correction was enabled for GC-content bias and chromosome-end bias. Smaller-scale CNVs were also evaluated by comparing the copy number at each position in evolved strains to the copy number in 2 sequenced ancestral strains. Positions elevated only in evolved strains were further examined individually, and the location mapped to the genome using the Candida Genome Database (candidagenome.org).

## Results

### Survival and growth ability in POS evolution

Replicate lines from 8 clinical strains of *C. albicans* were passaged with 1:1,000 dilutions every 24 or 72 h, for a total of 5 transfers in YPD + 0.5 µg/mL POS (what we refer to as POS0.5) and in YPD. We use the term “ancestral replicates” to indicate populations initiated from single colony replicates before evolution, and the term “evolved replicates” to refer to the replicates that survived the evolution experiment.

**Fig. 1.**
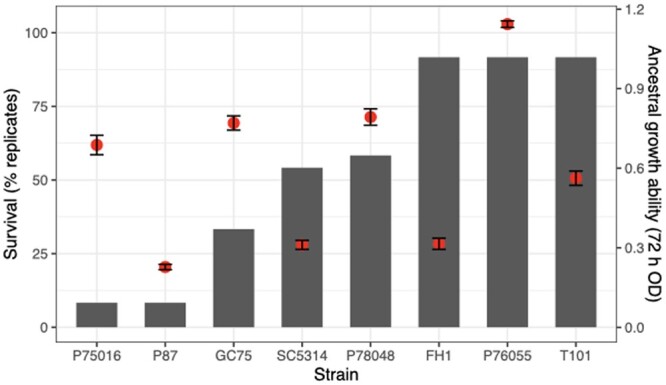
Differential survival of evolved replicates after 5 passages in POS0.5. The bars indicate the percentage of replicates that survived to the end. Dots indicate ancestral growth ability (optical density after 72 h in the evolutionary environment), shown on the right vertical axis. Each dot is the mean ± SE of 2 biological replicates.

**Fig. 2.**
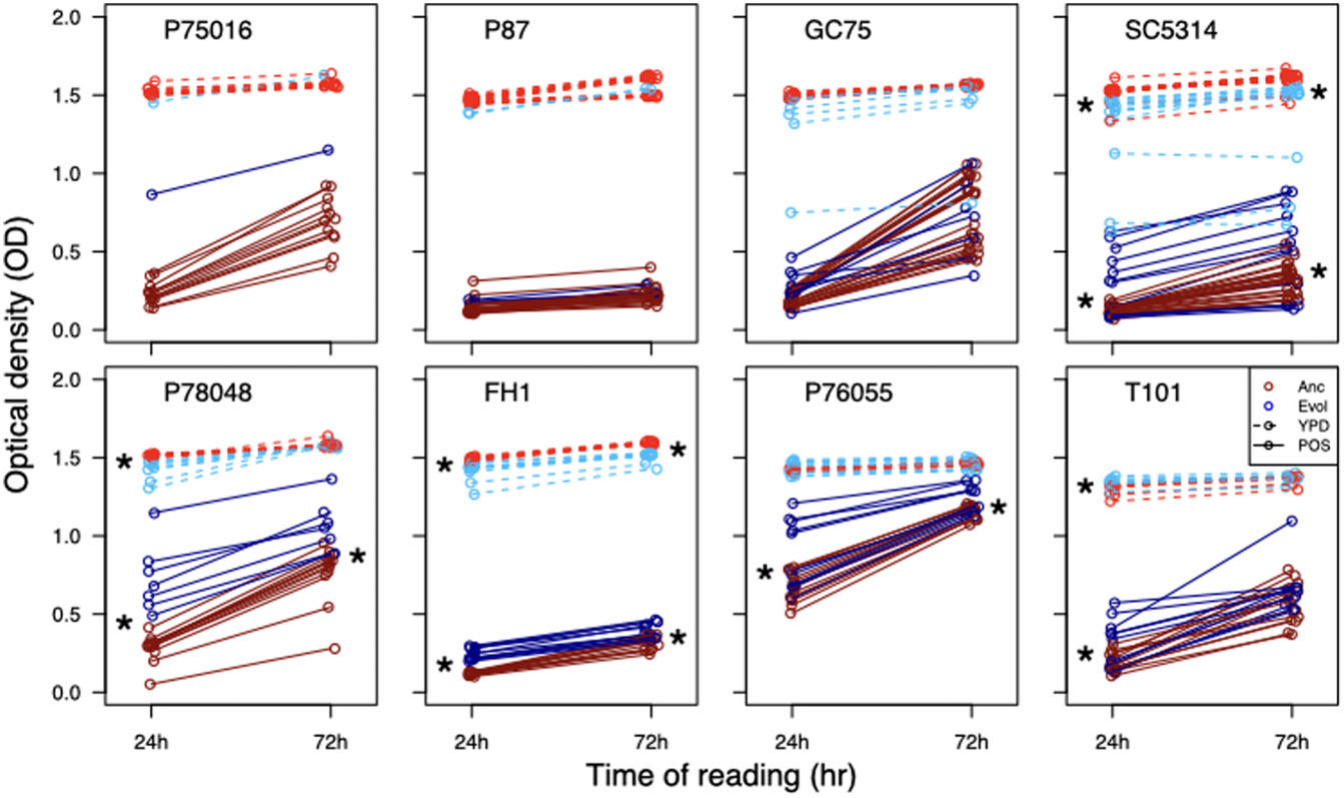
Fitness of ancestral and evolved replicates grown in YPD and POS0.5. Optical density was measured at 24 and 72 h. Shown here is only the replicates that were evolved through 72 h transfers in POS0.5. Evolved replicates grown in YPD are indicated with dashed lines, replicates grown in POS0.5 are full lines. Stars indicate statistical significance in a *t*-test comparing ancestral and evolved replicates at that time point (24 or 72 h of growth).

The evolutionary environment drug level was a strong selective pressure; the level of POS was at or above the MIC_50_ for all ancestral strains (CG75: 0.001; P87, P78048, SC5314: 0.002; P75016: 0.004; P76055: 0.016; FH1: 0.0625; T101: 0.5). The ancestral strains varied considerably in their ability to grow in POS0.5 within 24 and 72 h (i.e. the time between transfers; Figs. 1 and 2). No replicates survived in the POS0.5 24 h transfer experiment, while approximately half of the replicates (67 of 132) survived to the end of the POS0.5 72 h transfer experiment. Replicate survival to 72 h transfers in POS0.5 was not equal among strain backgrounds (Fig. 1). Surprisingly, ancestral growth ability in the evolutionary drug environment was not significantly correlated with the number of surviving replicates after evolution (Fig. 1; ancestral growth ability measured as optical density after 72 h of growth in POS, Pearson’s correlation, *t*_6_ = 0.91, *P = *0.40). This lack of correlation indicates that strain-specific genomic differences that influence something other than the ancestral growth ability in drug, likely correlate with evolvability to POS0.5. All replicate lines survived 24 and 72 h transfers in the standard rich medium YPD. The majority of the remaining analysis is based on the 72 h transfer experiment in POS0.5, except where indicated.

We measured the growth ability (optical density) of all ancestral and evolved replicates at 24 and 72 h in POS0.5. The 24 h OD readings should at least partially correlate with growth rate, as they reflect the achieved growth ability during or before exponential growth, while OD at 72 h reflects growth ability at the time of the transfers. Evolved replicates from the 5 strains with the highest number of surviving replicates (P78048, SC5314, FH1, P76055, and T101) had a higher growth ability than ancestral replicates after 24 h in POS (Fig. 2; *t*-test results in Table 1) and 4 of the 5 strains retained the advantage after 72 h of growth (Fig. 2, Table 1). There were not enough surviving replicates from P87 or P75016 to properly conduct statistical tests, though the one surviving replicate from P75016 also very clearly has an advantage in growth ability over the ancestral replicates. Evolved replicates from GC75 had no improvement over ancestral replicates at 24 or 72 h. Posaconazole evolved replicates from all strain backgrounds showed an inconsistent trade-off between improvement in growth ability in drug and reduction in growth ability in YPD, i.e. it was not consistently the case that the evolved replicates that improved the most in drug incurred the largest cost in YPD. There was no correlation between growth improvements in POS and growth reductions in YPD in any strain background at either 24 h (Pearson correlation tests; 24 h—P78048: *t*_5_ = 1.95, *P = *0.11; SC5314: *t*_11_ = 1.74, *P = *0.11; FH1: *t*_9_ = −0.97, *P = *0.36; P76055: *t*_9_ = 1.09, *P = *0.31; T101: *t*_9_ = 0.72, *P = *0.49) or at 72 h (P78048: *t*_5_ = −0.85, *P = *0.43; SC5314: *t*_11_ = 1.46, *P = *0.17; FH1: *t*_9_ = −1.11, *P = *0.29; P76055: *t*_9_ = −0.89, *P = *0.40; T101: *t*_9_ = 0.37, *P = *0.72). Evolved replicates from 3 strains (P78048, SC5314, and FH1) had a minor reduction in growth ability in YPD at 24 h, which continued to 72 h for SC5314 and FH1. T101 had a minor improvement in growth ability at 24 h in YPD. Of note, the magnitude of growth difference between statistically significant ancestral and evolved replicates tended to be considerably less in YPD compared to drug (Table 1).

**Table 1.** *T*-test results comparing optical density of ancestral and evolved replicates grown for 24 h (left) and 72 h (right) in POS (top) and YPD (bottom).

Strain	Evol-Anc	Statistic	Evol-Anc	Statistic
	POS 24 h		POS 72 h	
A03	0.08	*t*_7.9_ = −2.0, *P* = 0.09	−0.08	*t*_10.8_ = 0.8, *P* = 0.45
A04	0.5*	*t*_8.2_ = −5.4, *P* < 0.001	0.33*	*t*_15.0_ = −3.9, *P* = 0.002
A10	0.2*	*t*_13.5_ = −2.9, *P* = 0.013	0.09*	*t*_15.0_ = −3.0, *P* = 0.009
A12	0.12*	*t*_13.2_ = −2.3, *P* = 0.036	0.09	*t*_20.1_ = −1.5, *P* = 0.15
A17	0.17*	*t*_12.2_ = −2.9, *P* = 0.013	0.18*	*t*_13.6_ = −2.2, *P* = 0.048
A18	0.13*	*t*_11.5_ = −11.4, *P* < 0.001	0.08*	*t*_19.8_ = −4.3, *P* < 0.001
	YPD 24 h		YPD 72 h	
A03	−0.15	*t*_7_ = −1.65, *P* = 0.14	−0.12	*t*_7_ = 1.36, *P* = 0.22
A04	−0.09*	*t*_7.2_ = −3.5.6, *P* = 0.009	−0.01	*t*_13.9_ = 0.84, *P* = 0.42
A10	0.02	*t*_11.0_ = −1.3.6, *P* = 0.22	−0.002	*t*_11.2_ = 0.14, *P* = 0.89
A12	0.04*	*t*_20.6_ = −3.0.6, *P* = 0.007	0.01	*t*_20.7_= −1.16, *P* = 0.26
A17	−0.24*	*t*_12.3_ = 2.86, *P* = 0.014	−0.24*	*t*_12.3_ = 2.74, *P* = 0.018
A18	−0.08*	*t*_10.6_ = 4.25, *P* = 0.002	−0.09*	*t*_11_ = 9.05, *P* < 0.001

*p < 0.05.

### Widespread decreases in drug resistance and increases in drug tolerance in POS-evolved replicates

**Fig. 3.**
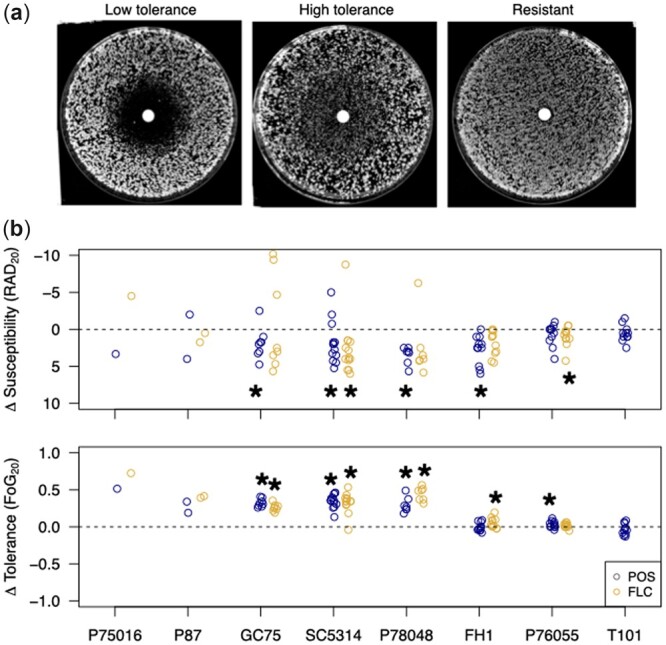
a) Representative plate images of low and high tolerance and resistance in a disk diffusion assay. b) Susceptibility (top) and tolerance (bottom) of POS0.5-evolved replicates assayed on POS and FLC disks. Shown is the difference in phenotype between the evolved replicate and the median of 12 ancestral replicates. A negative change in susceptibility in the evolved replicate indicates an increase in resistance, and the *y*-axis of the top panel is reversed to reflect this. Stars indicate a significant difference compared to the ancestral replicates from a *t*-test (*P* < 0.05).

Drug susceptibility was computationally quantified as the radius of inhibition on a disk diffusion plate (Gerstein *et al.* 2016). An increase in susceptibility (i.e. decrease in resistance) is observed when the evolved radius is larger than the ancestral radius. Surprisingly, replicates from 6 strain backgrounds evolved an increase in susceptibility (Fig. 3; GC75: *t*_7.6_ = −2.7, *P* = 0.029; SC5314: *t*_12.7_ = −2.25, *P* = 0.043; P78048: *t*_6.8_ = −7.7, *P* = 0.0001; FH1: *t*_13.0_ = −4.3, *P* = 0.0008; strains P75016 and P87 had too few replicates for statistical testing). Six replicates from 4 strains did deviate from the rest and acquired decreased susceptibility (increased resistance; P75016, GC75, SC5314, and P78048). Evolved replicates from the 2 strain backgrounds with the highest number of surviving replicates did not change in susceptibility (P76065: *t*_13.6_ = −1.4, *P* = 0.17; T101: *t*_15.4_ = 0.8, *P* = 0.42). The opposite result was seen for drug tolerance, which is computationally determined as FoG_20_, the fraction of growth between the disk and RAD_20_ (Gerstein *et al.* 2016, Fig. 3). Evolved replicates from 6 strain backgrounds increased in tolerance, while 2 of the strains with a higher number of surviving replicates did not change (Fig. 3; GC75: *t*_11.6_ = −14.5, *P* < 0.0001; SC5314: *t*_14.0_ = 12.63, *P* < 0.0001; P78048: *t*_6.1_ = −7.3, *P* = 0.0003; FH1: *t*_19.8_ = 0.6, *P* = 0.59; P76055: *t*_18.0_ = −2.2, *P* = 0.042; T101: *t*_20.1_ = 0.9, *P* = 0.50). Evolved replicates from across strain backgrounds more consistently increased in tolerance rather than resistance, suggesting that tolerance is either more evolvable than resistance or was the primary phenotype under selection during the in vitro evolution to POS0.5.

All evolved replicates were also assayed for the evolution of cross-resistance and/or cross-tolerance to the most common triazole, FLC. Results in FLC were fairly similar to POS; evolved replicates tended to increase in FLC susceptibility (decrease in resistance) yet increase in FLC tolerance. The median increase in tolerance among replicates was quite high and fairly similar in both POS and FLC for GC75 (POS: 34%, FLC: 26%), SC5314 (POS: 37%, FLC: 35%), and P78048 isolates (POS: 24%, FLC: 43%). Similar to POS, although a small number of replicates evolved an increased resistance to FLC, the majority of replicates in most backgrounds increased in susceptibility (Fig. 3; FLC resistance—GC75: *t*_7.1_ = −0.52, *P* = 0.62; SC5314: *t*_13.7_ = −2.51, *P* = 0.025; P78048: *t*_6.0_ = −0.93, *P* = 0.39; FH1: *t*_11.2_ = −1.9, *P* = 0.08; P76055: *t*_16.8_ = −2.6, *P* = 0.02; tolerance—GC75: *t*_13.2_ = −13.2, *P* < 0.0001; SC5314: *t*_13.5_ = −8.26, *P* < 0.0001; P78048: *t*_6.0_ = −12.7, *P* < 0.0001; FH1: *t*_11.2_ = −1.9, *P* = 0.09; P76055: *t*_18.8_ = 1.3, *P* = 0.21). The ancestral (and evolved) replicates of T101 are resistant to FLC and changes to FLC tolerance in evolved replicates cannot be examined in this framework.

We further subjected the 12 evolved SC5314 replicates to a panel of 5 additional antifungal drugs to look for cross-resistance and cross-tolerance: VCZ, a triazole like POS and FLC; CTR and MCZ, which are imidazoles; NYT, a polyene; and 5-FC, a fluorinated analogue of cytosine. Similar to what we found across strain backgrounds for POS and FLC, very few replicates exhibited decreased susceptibility (increased resistance) to any additional drugs (Fig. 5c). By contrast, nearly all evolved replicates showed increased tolerance to all 5 azole drugs examined and essentially no change in NYT or 5-FC. Hence, cross-tolerance to azole drugs arose rapidly and repeatedly following evolution to POS.

### Inconsistent changes in drug resistance and drug tolerance in YPD evolved replicates

Replicates that were evolved with 24 and 72 h transfers in YPD exhibited much smaller changes in resistance and tolerance, though some evolved strains did differ significantly compared to the ancestors (Supplementary Fig. 1, Supplementary Table 2). There was no apparent pattern between strain background and the specific evolved changes. Evolved replicates from strain P87 (a strain with few surviving POS replicates) in both the 24 and 72 h transfer experiments had significantly increased susceptibility. By contrast, evolved replicates from strain P76055 and T101, the 2 strains with the most surviving POS replicates, had significantly decreased susceptibility (in both experiments for strain P76055 while only in the 24 h transfers for strain T101). Evolved replicates from strain GC75 in the 24 h transfer experiment decreased in tolerance, while replicates from strain FH1 in the 24 h transfer experiment increased in tolerance.

### Widespread karyotypic changes after POS evolution

**Fig. 4.**
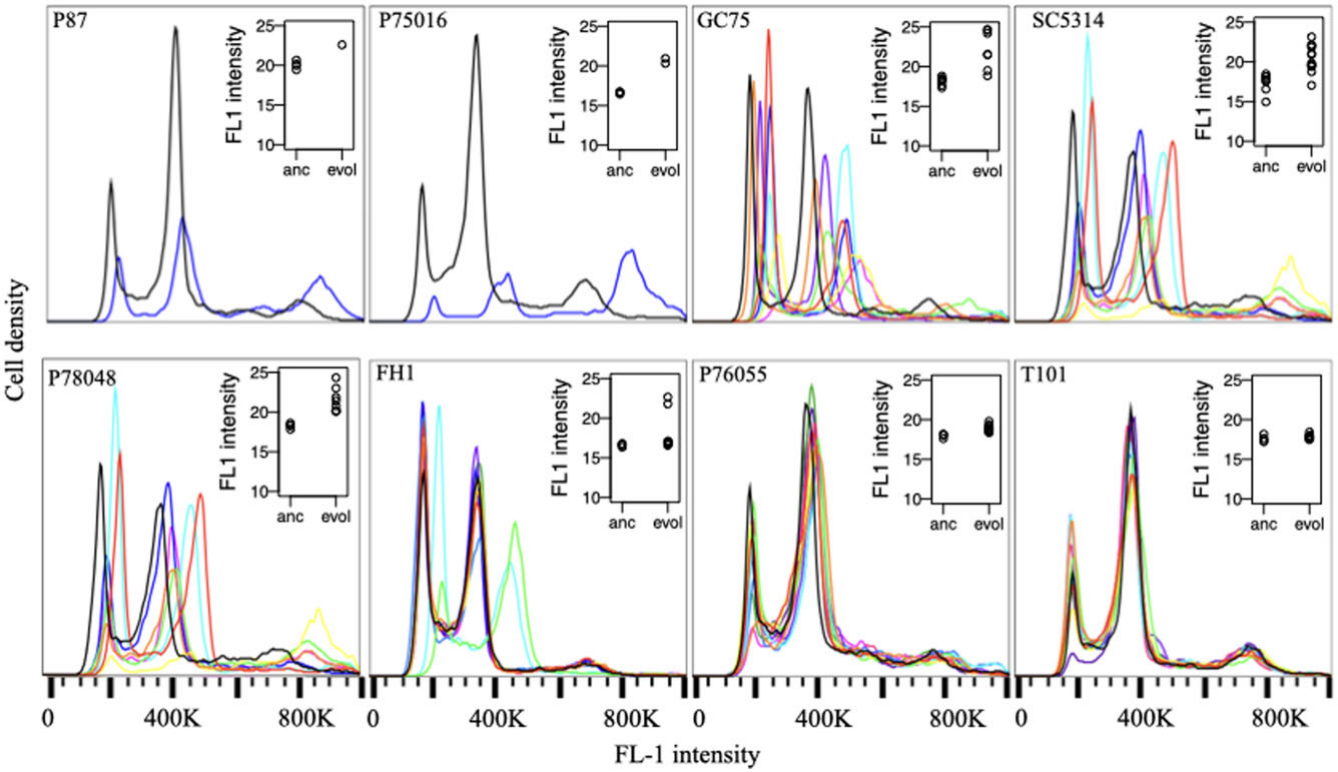
Flow cytometry traces from POS0.5-evolved replicates. Each trace is a single evolved replicate, the black trace in each panel is a diploid ancestral replicate. Inset panels display the mean G1 peak from each ancestral (anc) and evolved (evol) replicate for each strain.

Genome size changes in POS0.5-evolved replicates were examined with flow cytometry. Evolved replicates from the 3 strains with the highest proportion of surviving replicates (FH1, P76055, and T101) tended to retain genome sizes similar to the diploid ancestors, while the majority of evolved replicates from other strains varied in genome size (Fig. 4). Measured changes in genome size (G1 means) were generally consistent with aneuploidy rather than whole shifts in ploidy (Fig. 4, inset panels).

**Fig. 5.**
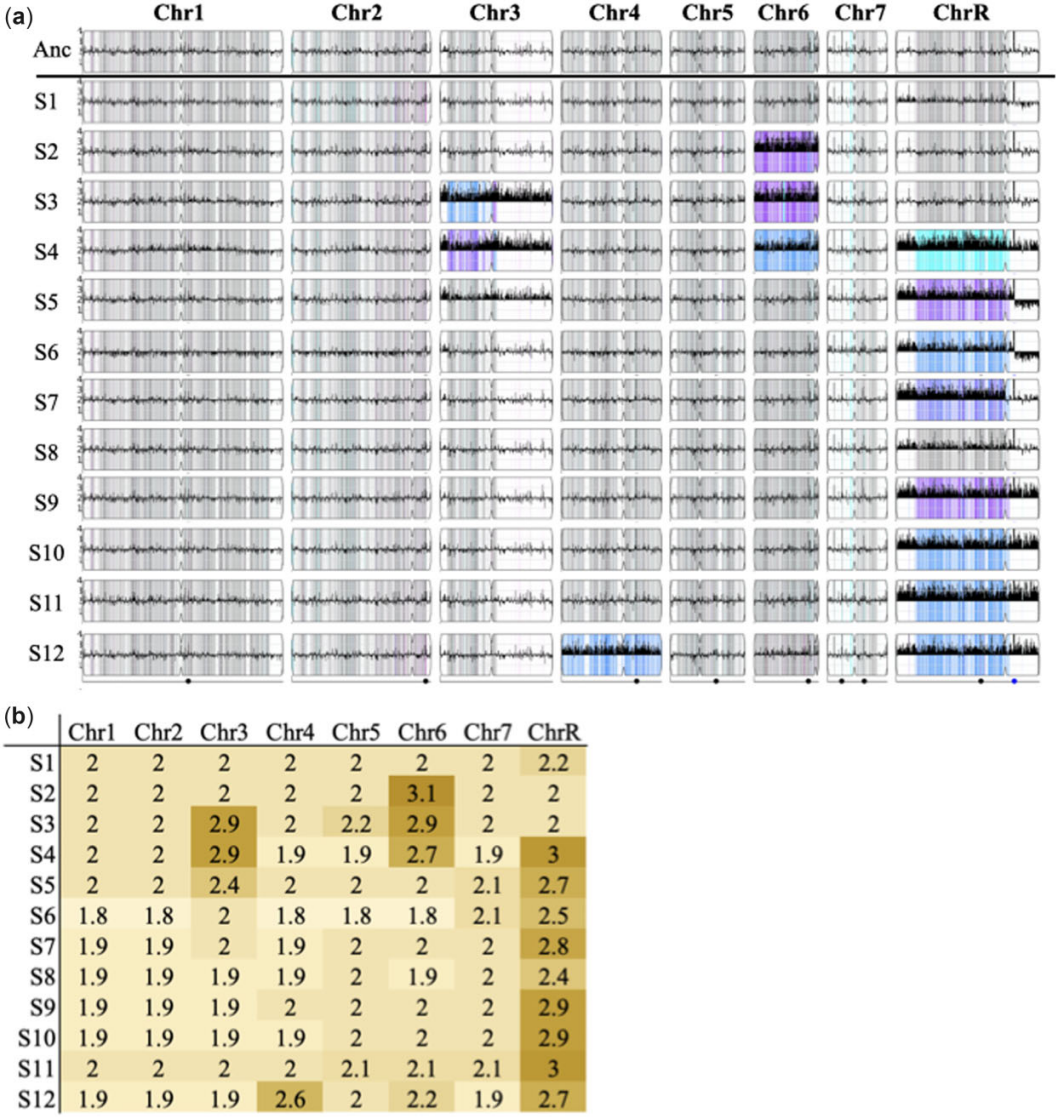
CNV in whole-genome sequenced replicates of SC5314. a) CNV and LOH in evolved strains from YMAP. Strains were compared to the SC5314 A21 reference. The density of heterozygous SNPs is shown as vertical lines spanning the height of each chromosome, with the intensity representing the number of SNVs in each 5-kb bin. Heterozygous SNVs are gray, while homozygous SNVs are colored based on the homolog that is retained: cyan for “AA,” magenta for “BB,” blue for “AAB,” and purple for “ABB.” White indicates ancestral LOH. CNV is shown as a black histogram drawn vertically from the center line spanning the chromosome. The *y*-axis is relative copy number. Centromere loci are illustrated as an indentation in the chromosome box. The dots on the bottom line indicate the positions of the major repeat sequences. b) Median copy number of all reads mapped to each chromosome.

To examine actual karyotypic changes and how they correlate with drug resistance and tolerance evolution, we performed whole-genome sequencing on 12 evolved replicates from SC5314. All but one evolved replicate had at least one trisomic chromosome. Trisomy of ChrR was the most common, with 9 replicates having either whole or partial trisomy of this chromosome. Of these 9, one replicate had additional aneuploidies in Chr3 and Chr6, and a second replicate had an extra copy of Chr4. Two additional evolved replicates also had an extra copy of Chr6, one alone and one in tandem with an extra copy of Chr3 (Fig. 5). We saw no apparent bias toward acquiring an extra copy of the A haplotype or the B haplotype. Most trisomies seem to have swept through the evolved populations, evidenced by a copy number close to 3 from population-level sequencing (Fig. 5b). In several cases, the measured copy number was between 2 and 3, likely indicating a polymorphic population, where some cells remained diploid (though we cannot rule out that some aneuploidies may be unstable and lost during the grow up from frozen evolved culture for sequencing). A small number of localized CNV were also present in evolved lines, but none have an obvious adaptive benefit (Supplementary Table 3). All small CNVs were either associated with long terminal repeats, major repeat sequences, telomeres, or existed as multiple sites on a single aneuploid chromosome in a single background, likely indicative of sequence mapping artifacts.

**Fig. 6.**
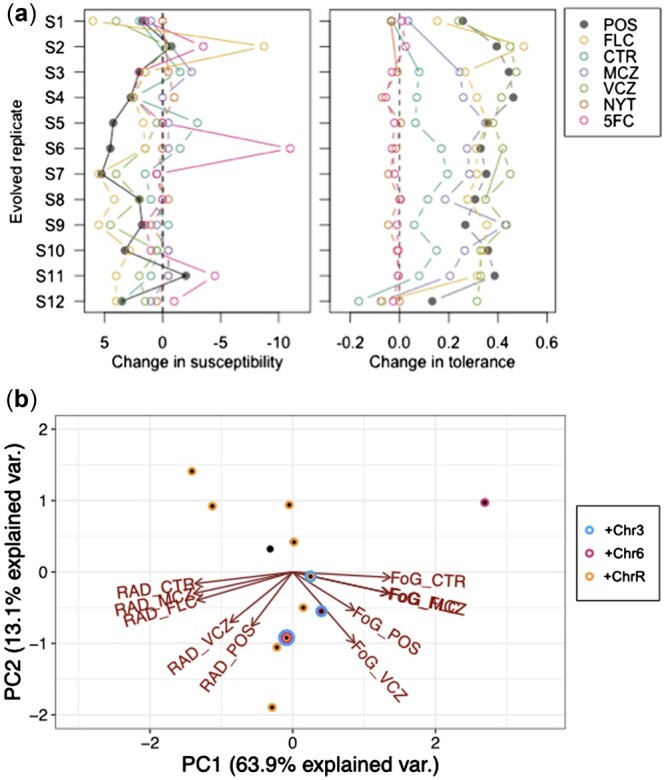
Cross-resistance and cross-tolerance in whole-genome sequenced replicates of SC5314. a) The change in susceptibility (left) and tolerance (right) in evolved replicates relative to the SC5314 ancestor. Susceptibility and tolerance were measured from disk assays for POS, FLC, CTR, MCZ, VCZ, NYT, and 5-FC. Note that a negative change in susceptibility is an increase in resistance. b) PCA plot to look for correlations between azole drug responses and the presence of aneuploidy. No clear clusters separate replicates with or without any of the 3 aneuploidies observed in multiple lines, indicating no clear correlation between azole resistance or tolerance and a specific aneuploidy.

We visually identified no clear phenotypic pattern to differentiate evolved strains with ChrR trisomy (or other trisomies) from other replicates. To statistically compare karyotypic differences to observed variation in azole drug resistance and tolerance, we conducted a principal component analysis of the 10 azole phenotypes (5 drugs assayed for resistance and tolerance). The top 2 principal components both had eigenvalues above 1 and combined to explain 77% of the variance. PC1 (63.9% of the variance, eigenvalue = 2.5) separated RAD and FoG measurements (Fig. 6b). PC2 (13.1% of the variance, eigenvalue = 1.1) differentiated among the different azole drugs, demonstrating that VCZ was most similar to POS (Fig. 6b). Plotting the 12 evolved replicates into this space demonstrated that there was no clear way to cluster the isolates when we look at any of the 3 aneuploidies that arose more than once. Hence, although repeated aneuploidy was observed, we cannot differentiate between the potential for multiple chromosomal aneuploidies to provide increased tolerance to POS0.5 (i.e. aneuploidy is beneficial) and the presence of aneuploidy as a consequence of azole drug exposure (i.e. aneuploidy is neutral).

We were similarly unable to link any mutations to the observed phenotypes. We conducted a targeted genomic analysis of 14 genes previously implicated in antifungal resistance: *TAC1*, *GSL2*, *CDR1*, *CDR2*, *ERG11*, *CRZ1*, *MRR1*, *UPC2*, *MDR1*, *RRP6*, *RIM9*, *NPR2*, and *GZF3*. We identified no high-confidence genic variants that differentiated evolved replicates from ancestral replicates except a single synonymous single-nucleotide variant (SNV) in *CDR2* (Leu1023Leu) in S11. The only other mutations in these genes were 7 LOH events in positions in S4 upstream and downstream of *GZF3* (at base pairs 646493, 646559, 646560, 646608, 647030, 649877, and 649963) that were interspersed with heterozygous positions (so not indicative of a large-scale LOH event).

## Discussion

Experimental laboratory evolution has been an effective method to study pathways of adaptation in a variety of biological contexts (Kawecki *et al.* 2012; Cooper 2018). Laboratory evolution of microbes at high population sizes (at times termed “experimental evolution” or “adaptive laboratory evolution”) is a particularly powerful way to examine parallelism and constraint in evolution at genomic and phenotypic levels (Cooper 2018; Gerstein and Sharp 2021). We evolved 12 replicates from 8 different strain backgrounds of the opportunistic human fungal pathogen *C.*  *albicans* to 0.5 µg/mL of the drug POS, with transfers every 24 or 72 h. This level of drug exceeds where we find robust growth in the absence of stress from most ancestral strains, and is an order of magnitude above the defined epidemiological cut-off value for MIC (Arendrup *et al.* 2011; CLSI 2020). As drug tolerance is defined as slow growth in the presence of high levels of drug, we reasoned that a more extended transfer period might select for drug tolerance to evolve. Unfortunately, none of the evolved replicates survived the 24 h transfers. In the 72 h transfer experiment, we also found high extinction (∼50% of the replicates).

Evolved POS0.5 replicates from the majority, but not all, strain backgrounds increased in their growth ability in the evolutionary level of drug. However, this did not lead to widespread increases in drug resistance, and the improvement of most replicates in the evolutionary environment was moderately low. Very similar results were found when a subset of these strains were evolved to a subinhibitory level of FLC (Gerstein and Berman 2020). A second evolution experiment done at the same subinhibitory level of FLC, however, did find ∼30% of replicates had increased resistance (Todd and Selmecki 2020). Another evolution experiment that paired a strong selective pressure with a long period of time between transfers (7 days) in caspofungin (a different class of drug), also found that many evolved diploid lines did not increase in resistance by the end of the 59-day experiment (Avramovska *et al.* 2021). In contrast, increased FLC resistance was evident in the earliest *C. albicans* evolution experiments, conducted in the T101 background in continually increasing levels of FLC for ∼330 generations (Cowen *et al.* 2000). Combined, these results suggest that in vitro evolution to antifungal drugs may not always result in increased drug resistance and that different suites of mutations may confer a benefit to different levels of the same drug.

The majority of surviving replicates did increase in POS tolerance and acquired cross-tolerance to FLC and other azole drugs. Very few studies have (yet) directly examined how different conditions influence how readily tolerance evolves in *C. albicans*. In an experiment where replicates were evolved with 72 h transfers to a subinhibitory level of FLC (i.e. a level of FLC below the MIC), changes in tolerance were also common, but both increases and decreases were observed (Gerstein and Berman 2020). We found a similar result here in YPD when replicates were transferred for 24 h: replicates from one strain background significantly increased in tolerance while replicates from a second significantly decreased. It seems clear that there is potential for drug tolerance to evolve quickly, which may have important clinical implications, particularly in cases of persistent candidemia infections when populations are exposed to drug stress for long periods of time, yet the underlying strain remains drug susceptible (Rosenberg *et al.* 2018). Additional experiments are required to tease apart how different drugs, the level of drug stress (subinhibitory vs inhibitory), and other specifics of environmental exposure drive differences in the propensity to acquire increases in drug resistance and drug tolerance.

Changes in genome size were also widely observed in our POS0.5-evolved replicates, similar to previous in vitro evolution experiments in FLC (Selmecki *et al.* 2006, 2009; Coste *et al.* 2007; Ford *et al.* 2015; Gerstein and Berman 2020, Selmecki *et al.* unpublished) and in strains passaged through a mouse GI model (Ene *et al.* 2018). Aneuploidies identified in FLC evolved strains typically involve genes known to be involved in FLC resistance mechanisms such as Hsp90, efflux pumps, and multidrug transporters *MRR1*, *CDR1*, *CDR2*, *CRZ1* on Chr3 (Mount *et al.* 2018; Todd and Selmecki 2020) and *ERG11*, *TAC1*, and calcineurin genes on Chr5 (Selmecki *et al.* 2008). In contrast, in our SC5314 evolved isolates, we predominantly found extra copies of ChrR (9 isolates), Chr6 (3 isolates), and Chr3 (2 isolates, in tandem with Chr6 aneuploidy in both cases). These aneuploidies were concurrent with increases in tolerance to POS (and other azoles), but not increases in resistance to POS or other tested drug classes. This is somewhat surprising, as a previous study in a different strain background found that an extra copy of ChrR increased resistance to FLC, KCZ, and MCZ (Li *et al.* 2015). Chr3 aneuploidy was previously identified to confer an increase in FLC tolerance, due at least in part to an extra copy of the urea transporter *NPR2* (Mount *et al.* 2018). Interestingly, Chr6 and ChrR were previously found to be the 2 most common aneuploidies remaining in otherwise euploid strains after 28 days of daily passaging of initially tetraploid and initially aneuploid strains in standard lab YPD (Hickman *et al.* 2015). This suggests that although it is possible there is a direct link between these specific aneuploidies and the increase in POS tolerance we observed, it could also be that these chromosomes carry genes that are beneficial in the context of in vitro evolution in general, or perhaps they simply carry the lowest cost in an otherwise euploid background in an environment where aneuploidy frequently occurs. Additional work is clearly required to definitively link aneuploidy to phenotype. Changes in chromosome copy number are frequently observed in experimental studies of fungal microbes, yet they are often observed without identifying a directly mechanistic link to fitness (Gerstein and Sharp 2021).

The rapid evolution of tolerance and cross-tolerance through in vitro evolution has not been previously observed in azoles nor linked to aneuploidy. Todd and Selmecki (2020) did, however, identify 3 isolates evolved to FLC in an in vitro experiment that had increased copy number of a subset of efflux pump, multidrug transporters, and stress response genes on Chr3, leading to increased tolerance and resistance to several azoles. Although the mechanism(s) that underlie drug tolerance are still being resolved, there are putative tolerance-associated genes on ChrR to target for follow-up studies. Transcriptomics and phenotypic analyses demonstrated that all Rim proteins (including *RIM9*, on ChrR) were important for FLC tolerance (Garnaud *et al.* 2018). Also intriguing and worthy of further study is *GZF3*, a GATA-type transcription factor of unknown function, one of 2 genes (alongside *CRZ1*) identified in an overexpression screen of 572 genes for FLC tolerance (Delarze *et al.* 2020). Tolerance has both a genetic component, likely involving genes in membrane biosynthesis/integrity and the stress response pathways (Cowen *et al.* 2014; Rosenberg *et al.* 2018; Berman and Krysan 2020; Todd and Selmecki 2020) and an environmental component, as growth conditions have been linked to the degree of tolerance exhibited (Gerstein *et al.* 2016; Berman and Krysan 2020). POS is not a substrate for *MDR1* or *FLU1* encoded efflux pumps (Chau *et al.* 2004; Hof 2006) hence there are likely to be both pan-azole and azole-specific genetic mechanisms underlying this complex trait.

Experimental evolution studies have contributed a wealth of knowledge toward understanding a wide range of factors that influence evolutionary dynamics. We used an in vitro evolution framework at a high level of the second-generation azole drug POS to evolve replicates from diverse *C. albicans* strains. We found that the survival of replicates was strongly dependent on strain background. The majority of evolved replicates improved in rapid growth in the evolutionary level of drug, yet very few replicates increased in POS resistance. Strain backgrounds with fewer surviving replicates were more likely to increase in POS drug tolerance and more likely to increase in genome size. Three chromosomal aneuploidies were observed in parallel in multiple evolved lines; these are largely different from those that have been shown to confer an increase in resistance to the related azole drug FLC, indicating that the genetic pathway to acquiring POS tolerance is distinct. As the azole drugs are fungistatic rather than fungicidal, further work is required to determine whether increases in tolerance upon repeated exposure to drugs are common in a clinical setting and whether tolerance is a stepping-stone in the path to resistance or represents a distinct peak in an adaptive trajectory.

## Data availability

Raw data and R scripts used for statistical analyses and to generate figures are available at https://github.com/acgerstein/posaconazole-evolution

Supplemental material is available at *G3* online.

## Supplementary Material

jkac156_Supplementary_Data
