# Supplementary figures and images for: Acquisition of cross-azole tolerance and aneuploidy in Candida albicans strains evolved to posaconazole
Source: G3 (Bethesda). 2022 Jul 26;12(9):jkac156. doi: 10.1093/g3journal/jkac156 (PMC9434289; doi:10.1093/g3journal/jkac156)

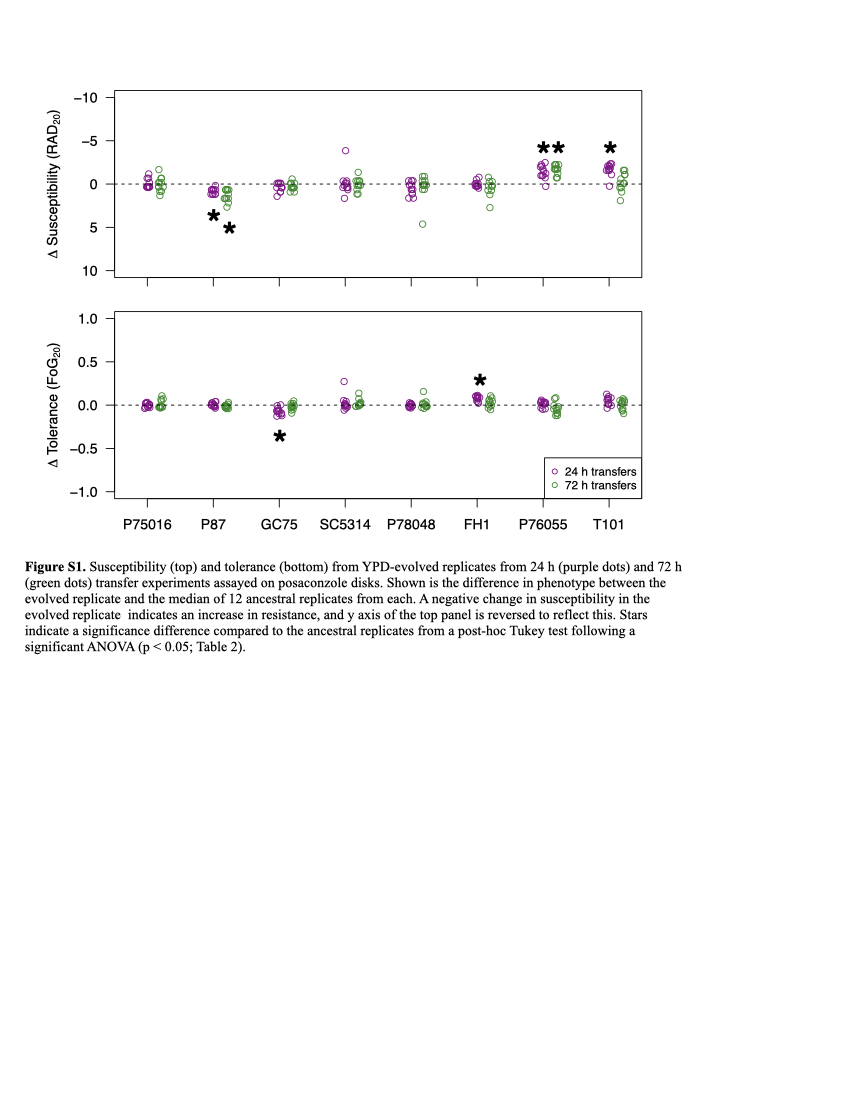

Supplement: jkac156_Supplementary_Data [file jkac156_supplementary_data.zip › jkac156_Supplementary_Figure_S1.png]
